# Combining Motifs, CRE Activity, And Gene Expression Data Using ML Greatly Improves the Accuracy of Tissue-Specific TF Network Maps

**DOI:** 10.1101/2025.10.10.681634

**Published:** 2025-10-13

**Authors:** Wooseok J. Jung, Sandeep Acharya, Daniel P. Ruskin, Shu Liao, Vaha Akbary Moghaddam, Zolboo Erdenebaatar, Michael R. Brent

**Affiliations:** 1 Department of Computer Science and Engineering, Washington University, St Louis, MO; 2 Division of Computational and Data Sciences, Washington University, St Louis, MO; 3 Division of Statistical Genomics, Washington University School of Medicine, St Louis, MO; 4 College of Information, University of Maryland, College Park, MD

## Abstract

Transcription factor (TF) network maps link TFs to their direct, functional gene targets whose transcription they regulate by binding cis-regulatory elements (CREs). Existing methods to reconstruct these networks typically rely either on TF motifs in CREs or gene expression data alone, limiting their accuracy. Motif data alone often fail to identify actual TF binding sites, while expression data cannot distinguish direct from indirect regulatory relationships. Additionally, accurate TF networks must be tissue-specific due to varied TF activities and expression patterns across tissues.

We introduce METANets (Motif Expression TF Association Networks), a novel supervised ensemble learning approach integrating TF motifs, TF binding locations, CRE activity, and gene expression data. Using XGBoost models, we predict TF binding in CREs based on TF motif and gene expression features, derived from linear (LASSO) and non-linear (BART) regression models trained on tissue-specific and aggregated RNA-seq data. This approach was applied to 36 human tissues from GTEx.

METANets significantly outperform existing motif-only and expression-only approaches, capturing more direct, functional TF targets. Evaluations against ChIP-seq binding data and gene ontology enrichment demonstrate METANets’ superiority in identifying functional targets directly bound by TFs. Furthermore, tissue specificity assessed through tissue-specific expression quantitative trait loci (eQTLs) confirms METANets effectively capture tissue-specific regulation, performing comparably with other networks.

Our approach markedly improves TF network reconstruction by combining complementary data types, enhancing the accuracy and utility of tissue-specific transcriptional regulatory maps. METANets provide robust resources for researchers investigating TF-mediated regulation within human tissues.

## Background

A transcription factor (TF) network map is a directed graph in which nodes represent TFs and genes, and edges link TFs to the genes they directly regulate. Numerous algorithms have been developed for mapping TF networks from a variety of data types, and these methods generally fall into two broad categories. The first category uses TF binding evidence, such as DNA sequence motifs [1] or TF ChIP-seq [2], often combined with promoter and enhancer annotations to define regulatory elements. Motif data describe locations where a TF could bind and TF ChIP-seq data reveal the genomic regions where a TF actually binds. However, binding evidence alone cannot confirm whether a TF functionally affects the transcription rates of its target genes. For instance, many TF-bound motifs have no apparent effect on gene transcription [3–5]. Furthermore, most motifs for a TF are not bound by the TF [6, 7], and many sites where a TF binds lack motifs [8, 9]. Another class of methods uses gene expression measurements under baseline conditions or after perturbations of TF expression [10–15]. While perturbation-response data can reveal the functional effects of TFs, they do not confirm direct binding. Moreover, expression-based methods are prone to capturing indirect effects, as correlations between TF and target gene (TG) expression may arise from transitive effects such as shared upstream regulators or mediated interactions [16–18].

While many regulatory relationships are shared across tissues, some TF-TG interactions are context-specific. TF network maps inferred from gene expression and binding data have been shown to vary significantly across cell types and tissues [19–22]. Our group has previously mapped TF networks in *Saccharomyces cerevisiae* and *Drosophila melanogaster* using both TF binding and gene expression data, successfully capturing direct and functional targets of TFs [11, 12]. However, these mapping efforts focused on a single, aggregate context and did not address regulatory variation across tissues, which limits their utility for understanding context-specific gene regulation in human tissues. Most widely used approaches for tissue-specific TF network mapping remain limited in their ability to capture direct and functional targets. For example, TF motif-based approaches using cis-regulatory element (CRE) activity levels across tissues can suggest tissue-specific binding potential, but do not confirm functional relationships [23], while correlation-based networks fail to account for the physical binding of the TF in the target gene’s CREs [24, 25]. One notable exception is [20] which uses PANDA, combining TF motifs and protein-protein interactions (PPI) with tissue-specific expression data in a message-passing framework, to map 38 tissue-specific TF networks. Building on our prior work, we introduce METANet, a supervised machine learning approach to predict tissue-specific direct and functional TF-TG regulatory relationships. Unlike PANDA’s unsupervised approach, METANet uses supervised learning which allows the model to learn directly from TF ChIP-seq-based binding signals. Using tissue-specific gene expression data and TF network maps derived from [23] as predictor variables, METANet predicts the probability that a TF binds a target gene in TF-ChIP-seq data the model has not seen.

This paper makes four contributions. We provide a comprehensive resource of human tissue-specific TF network maps across 36 tissues, encompassing ~237 TFs and ~12,150 protein-coding genes. We evaluate the ability of our TF network maps to capture direct and functional targets of TFs using four evaluation metrics and compare our performance to the networks proposed by Sonawane et al. We also compare to the original networks from Marbach et al (which does not use gene expression data). Across 36 tissues, we show that METANet better captures both the direct and functional targets of TFs compared to benchmark network maps. We also found that the tissue-specificity of METANet is comparable to that of the network maps from Marbach et al and Sonawane et al. To explore potential applications of METANet, input it to FISHNET [26], a network-driven gene prioritization method, using transcriptome-wide association (TWAS) summary statistics for traits associated with cardiovascular risk from the Long Life Family Study (LLFS) cohort. METANet identified replicated gene-trait associations that were missed when other networks were passed as inputs to FISHNET. Furthermore, METANet identified regulatory mechanisms that may mediate the effect of known trait-associated variants.

## Results

### Overview of TF Network Map Reconstruction

**Fig. 1:**
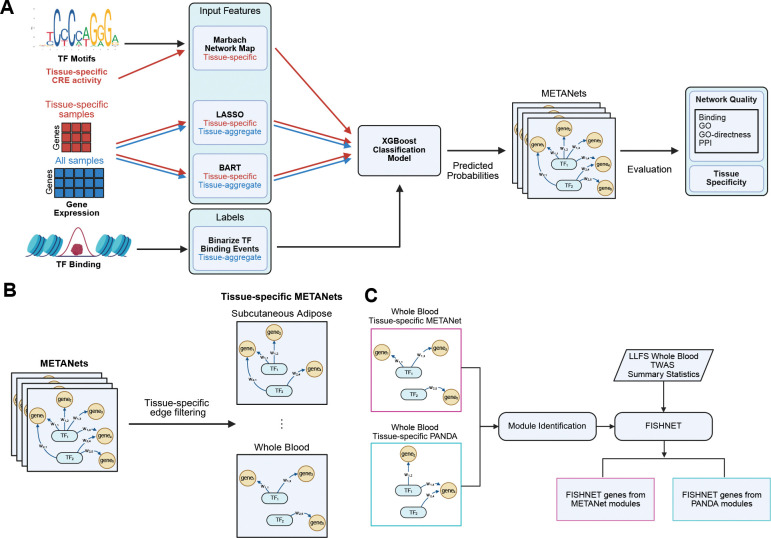
METANets overview (**A**) For each tissue, METANets combines evidence scores from tissue-specific TF network maps from Marbach et al. 2016, tissue-specific and tissue-aggregate LASSO and BART regression models from GTEx expression data, and binarized TF binding labels using XGBoost. The predicted probabilities from the XGBoost model are used as edge scores in our tissue TF network maps. Red arrows indicate tissue-specific data while blue arrows indicate tissue-aggregate data. The predicted METANets are evaluated for network quality and tissue specificity. (**B**) Tissue-specific METANets are inferred from the predicted METANets by filtering for tissue-specific edges. (**C**) Modules identified from tissue-specific METANet and tissue-specific PANDA are used in FISHNET with TWAS summary statistics from the LLFS to identify FISHNET genes.

To map tissue-specific transcription factor (TF) regulatory networks, we developed METANets, a supervised learning framework that integrates TF motif data, gene expression-derived features, and TF binding evidence. For each of 36 human tissues, we trained an XGBoost model [27] to predict whether a TF binds within the cis-regulatory elements (CREs) of a gene, using ChIP-seq data from REMAP 2020 [28] as ground truth (Fig. 1). Each instance in the model corresponds to a candidate TF-target gene (TG) pair and was represented by five biologically motivated features: (1) a motif-based regulatory score from the tissue-specific Marbach network [23], which combines TF motif occurrences with tissue-specific CRE activity; (2–5) four expression-derived features capturing both linear and nonlinear TF-TG relationships. The features were derived by regressing the expression of each gene on the expression of all TFs using both LASSO [29] and Bayesian Additive Regression Trees (BART) [30]. We extracted from the model for each gene measures of the relatedness of each TF’s expression to the gene’s expression (see Methods for details). Both regression algorithms were applied to both tissue-specific and tissue-aggregated RNA-seq data from GTEx [31] (Fig. 1). We used 10-fold nested cross-validation to train and evaluate each model. Predicted probabilities from test folds were used as edge scores in the METANet, linking TFs to their likely direct functional targets. In total, we mapped the TF networks of 36 human tissues, encompassing an average of 237 TFs and 12150 genes.

### METANets Capture Direct and Functional Targets of TFs

We compared METANet against the motif-based Marbach networks, constructed using motif occurrences and tissue-specific CRE activity levels [23], and PANDA networks from Sonawane et al., built using motifs and expression via an unsupervised approach [20], across 26 common tissues. For the PANDA networks, we used the fully connected PANDA output networks from Sonawane et al. prior to filtering for tissue-specific edges. All networks we evaluated include continuous edge scores, from which discrete networks can be created by thresholding and retaining only edges above the chosen cutoff. To enable fair comparisons across tissues, we applied thresholds scaled to the average number of targets per TF (e.g. top 20000 edges for 200 TFs implies 100 targets per TF), and we examined how evaluation results changed as more, lower-scoring edges were included.

To assess network quality, we used four complementary metrics: Binding, Gene Ontology (GO), GO-directness, and protein-protein interaction (PPI). To provide an empirical null expectation for each metric, we generated 50 random networks by permuting the edge scores of METANets. These random networks were also subjected to the same cutoff thresholds as other networks before evaluation.

#### Binding

**Fig. 2:**
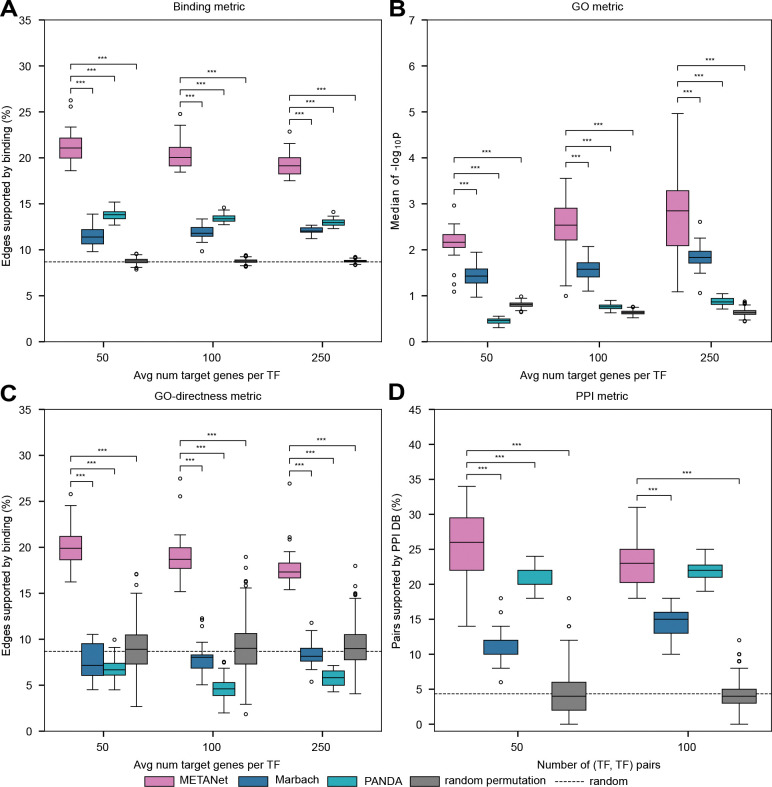
Evaluation of network quality. Performance of METANets, Marbach networks, and PANDA networks on 26 tissues for which all three networks were available. Pink: METANets; Blue: Marbach network maps; Cyan: PANDA network maps; Gray: random expectation based on permutations of METANets; black dashed: random expectation. (**A**) Binding metric (defined in main text) calculated at different edge-score thresholds. METANets outperform all other network maps. Random expectation is the probability that a randomly selected TF-TG edge will be supported by binding data. (**B**) GO metric (defined in main text). METANets outperform all other network maps and the random expectation. Random permutation is the GO metric for 50 METANets in which edge scores were randomly permuted. (**C**) GO-directness metric (defined in main text). METANets significantly outperform all other network maps. Random expectations include the GO-directness evaluations of the GO results of the 50 permuted network maps generated in (B) and the binding metric random expectation. (**D**) PPI metric (defined in main text). METANets significantly outperform all other network maps. The random expectation is the probability that a randomly selected (TF, TF) pair will be supported by PPI data. Significance bars only drawn for significant paired *t-*tests with METANet. *P≤0.05, **P≤0.01, ***P≤0.001.

The Binding metric is the fraction of edges supported by binding data showing that the TF binds in one or more of the target’s CREs, sourced from the FANTOM5 database [32, 33]. METANet significantly exceeded both Marbach and PANDA (paired *t*-tests, P<0.001 for each; Fig. 2A) and far exceeded the random expectation (*t*-test, P= 1.8 × 10^−32^), indicating stronger enrichment for directly bound targets.

#### Gene Ontology (GO) enrichment

To compute the GO metric, we carried out over-representation analysis of GO biological process annotations among the targets of each TF. Each TF’s score was the maximum −log P-value over all GO terms. If a TF’s target set showed no significant enrichment for any GO biological process, we assigned the TF a P-value of 1 (equivalent to −log P = 0). The final network-level score was taken as the median of these scores across all TFs. METANet outperformed Marbach and PANDA (both P<0.001; Fig. 2B) and surpassed the random permutation (P<0.001; see Methods), showing greater functional coherence among the predicted targets of each TF.

#### GO-directness

The GO-directness metric is the fraction of targets annotated with each TF’s most significant GO term that are also supported by ChIP-seq binding. This metric penalizes networks that achieve high GO scores by including many, functionally related, but indirect, targets. METANet retained significantly more binding support than both Marbach and PANDA (P<0.001; Fig. 2C) and the random permutations (P<0.001).

#### Protein-protein interaction (PPI)

The PPI metric is the fraction of top-ranked (TF, TF) pairs, based on target set similarity, that are supported by high-confidence physical protein-protein interactions in the STRING database (confidence score ≥ 0.7) [34]. METANet outperformed Marbach (P<0.001) and PANDA (P<0.05), recovering biologically corroborated physical TF-TF interactions (Fig. 2D).

Together, these results show that METANets are enriched for direct and functionally coherent TF-TG interactions relative to other networks.

### Combining Motif and Expression Features Improves Network Mapping

**Fig. 3:**
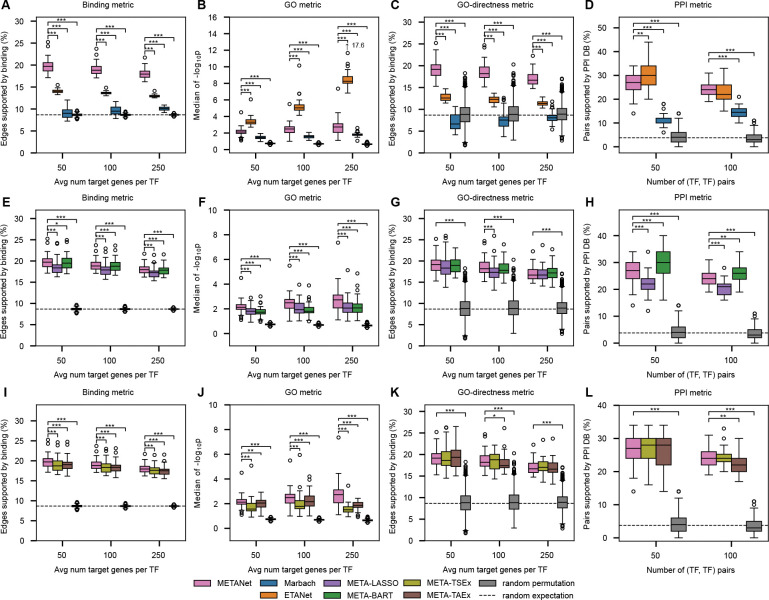
Network quality evaluations against ablated networks. (**A-B**) Performance of METANets and the Marbach networks (motif-only) and ETANets (expression-only) on 36 tissues. (**A**) Binding metric. METANets significantly outperformed both ETANets and Marbach networks. (**B**) GO metric. ETANets outperformed METANets and Marbach networks. (**C**) GO-directness metric. METANets outperformed ETANets, predicting more accurate TF network maps of direct and functional TF-TG interactions. (**D**) PPI metric. ETANets outperformed Marbach networks in both thresholds but were comparable to METANets for top 100 (TF, TF) pairs. **(E-H)** Performance of METANets and ablated TF network maps META-LASSO and META-BART. (**E**) Binding metric. METANet and META-BART significantly outperform META-LASSO, while METANet and META-BART perform comparably. (**F**) GO metric. METANets outperforms both ablated TF network maps. (**G**) GO-directness metric. All three TF network maps significantly outperform the random expectation based on 50 permutations of METANets. METANets is comparable with both ablated network maps. (**H**) PPI metric. META-BART outperforms both METANets and META-LASSO. **(I-L)** Performance of METANets and ablated TF network maps META-TSEx and META-TAEx. (**I**) Binding metric. METANets significantly outperform both ablated TF network maps. (**J**) GO metric. METANets outperforms both ablated TF network maps. (**K**) GO directness metric. All three TF network maps significantly outperform the random permutation and METANets is comparable with both ablated network maps. (**L**) PPI metric. All three network maps perform similarly with and outperform the random permutation. Significance bars only drawn for significant paired *t*-tests with METANet. *P≤0.05, **P≤0.01, ***P≤0.001.

We next evaluated the contribution of TF motif- and gene expression-derived features by comparing METANets to the Marbach networks, which are built using only motifs, and to ETANets, which we built in the same way as METANets but not including the edge score from Marbach networks, using only expression data across all 36 tissues. METANets significantly outperformed both Marbach and ETANet in the Binding metric (P<0.001), indicating the value of integrating both information sources (Fig. 3A). Interestingly, ETANet performed best in the GO metric, suggesting that gene expression alone captures broader functional coherence (Fig. 3B). However, METANets significantly outperformed ETANets in the GO-directness metric (P<0.001), indicating that many of ETANets’ additional functionally coherent targets are likely indirect (Fig. 3C). Thus, METANets provide a more accurate map of direct, functional TF-TG interactions than both the Marbach networks and the PANDAs networks.

### Linear and Nonlinear Expression Features are Synergistic

To assess whether combining linear (LASSO) and nonlinear (BART) expression-derived features improved network quality, we compared METANet against ablated versions using only one feature type: META-LASSO and META-BART. In the Binding metric, METANet and META-BART outperformed META-LASSO. Compared to each other, METANet and META-BART performed comparably (Fig. 3A). In the GO metric, METANet significantly outperformed both ablated network maps (Fig. 3B), suggesting that leveraging both LASSO and BART features better captures functionally coherent relationships. GO-directness was similar across models (Fig. 3D), consistent with gains in functional targets without loss of direct targets.

### Combining Tissue-specific and Tissue-aggregate Expression Features is Helpful

To assess the contribution of expression-derived feature specificity, we compared METANet to two ablated variants. META-TSEx retains the motif-based Marbach feature and tissue-specific expression features and excludes tissue-aggregate expression features (LASSO and BART trained on all samples pooled across tissues). Conversely, META-TAEx retains the Marbach feature and tissue-aggregate expression features, but not the tissue-specific expression features. In the Binding and GO metrics, METANet outperformed both ablated models (Fig. 3E, F). In the GO-directness metric, METANet significantly outperformed META-TAEx at one threshold (100 targets per TF) (Fig. 3G). Together, these results show that combining both tissue-specific and tissue-aggregate expression features enables METANets to identify substantially more functionally coherent targets of TFs without sacrificing direct binding support.

### METANets are Tissue Specific and Comparable to Existing Methods

We evaluated tissue specificity using tissue-specific expression quantitative trait loci (eQTLs) from GTEx [35]. For each edge in each METANet, we queried whether at least one tissue-specific eQTL overlapped the TF’s ChIP-seq peaks in the promoter or annotated enhancers of the target gene. We defined two complementary metrics:

1. eQTL support: Fraction of edges supported by at least one tissue-specific eQTL.
2. eQTL count: Sum, across all edges, of the number of tissue-specific eQTLs supporting each edge.

To assess a network’s tissue-specificity, we performed a permutation test. First, we calculated an observed statistic $S_{\mathrm{obs}}$ by ranking all 36 networks within each tissue and summing the ranks of the “matching” pairs (e.g., the rank of the liver network in the liver tissue). A smaller sum indicates better overall specificity. Next, to generate a null distribution, we performed 10000 permutations. In each permutation, we randomly shuffled the network labels before summing the ranks of the newly assigned matching pairs to obtain the permutation statistic $S_{\mathrm{perm}}$. This process simulates the null hypothesis that there is no true correspondence between a network and its native tissue. The empirical p-value was then calculated as the proportion of permutations with $S_{\mathrm{perm}}\leq S_{\mathrm{obs}}$.

### METANets are as tissue specific as the Marbach networks

We compared the tissue specificity of top-scoring edges in METANets and the Marbach networks. METANets showed significant tissue specificity compared to the null in both eQTL support and eQTL count (P<0.05) at higher average targets per TF thresholds (≥100 targets per TF) (Table 1). The Marbach networks showed similar patterns, and the Wilcoxon signed-rank test revealed no significant difference in the distributions of matching tissue ranks between METANets and Marbach networks in tissue specificity (Table 3). Thus, METANets achieve comparable tissue specificity while outperforming the Marbach networks in all network quality metrics.

### METANets Comparable to PANDA in Tissue Specificity but Outperform in Network Quality

Against the PANDA networks (26 tissues), neither PANDA nor METANets showed significant tissue specificity in eQTL support (Table 2). METANets were significantly better than chance in eQTL count in one threshold (average 100 targets per TF), but PANDA was not. The Wilcoxon signed-rank test found no significant difference between METANets and PANDA in either metric, indicating comparable tissue specificity (Table 4).

It is important to note that PANDA was originally developed as a general framework for inferring regulatory networks by integrating motif, expression, and protein-protein interaction data [36]. In its original form, PANDA produces a fully connected, weighted TF-gene network, but does not explicitly model tissue-specificity. The concept of *tissue-specific* PANDA networks was introduced by Sonawane et al. (2017), who applied PANDA to 38 GTEx tissues and then post-processed the resulting networks with a tissue specificity filtering step. In this approach, each edge’s weight in a given tissue was compared to its distribution across all tissues, and a normalized interquartile range (IQR) score was used to identify edges unusually strong in one tissue. Edges with high IQR scores were retained as “tissue specific.”

To enable direct comparison with the tissue-specific filtering strategy of Sonawane et al., we applied the same IQR-based approach to METANets. These filtered networks were evaluated without thresholding for top-scoring edges, as the number of TFs, genes, and edges varied drastically across tissues. Tissue-specific METANets showed significant eQTL support but not eQTL counts, while tissue-specific PANDA networks showed no significant signal in either metric (Table 5). Again, no significant difference was found between tissue-specific versions of METANets and PANDA networks (Wilcoxon signed-rank P > 0.05) (Table 6).

**Fig. 4:**
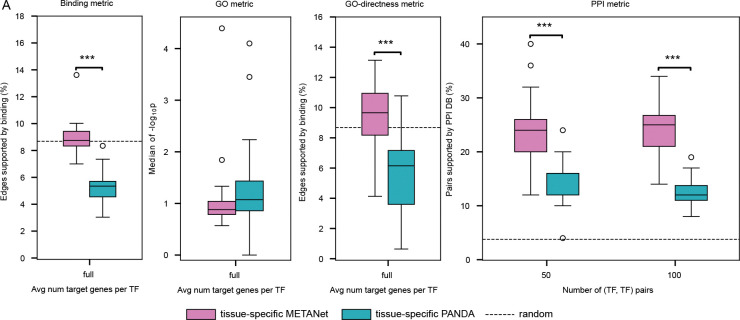
Network quality evaluation of tissue-specific networks **(A-D**) Network quality evaluation of tissue-specific METANets and tissue-specific PANDA networks. (**A**) Binding metric. Tissue-specific METANets significantly outperformed tissue-specific PANDA networks (paired *t*-test, P<0.05). (**B**) GO metric. No significant difference between tissue-specific METANets and tissue-specific PANDA networks. (**C**) GO-directness metric. Tissue-specific METANets outperformed tissue-specific PANDA networks (P<0.001). (**D**) Tissue-specific METANets outperformed tissue-specific PANDA networks at thresholds of 50 and 100 (TF, TF) pairs. *P≤0.05, **P≤0.01, ***P≤0.001.

Despite similar performance in tissue specificity, tissue-specific METANets outperformed tissue-specific PANDA in the Binding and GO-directness metrics (P<0.05 and P<0.001, respectively), while performing similarly in the GO metric (Fig. 4A–C). Tissue-specific METANets also performed better in the PPI metric overall (Fig. 4D).

### Tissue-specific METANets are Useful for Genetic Analyses

To evaluate the biological relevance of tissue-specific METANets, we applied FISHNET, a method that uses prior biological knowledge to identify genes with suggestive association signals that are more likely to replicate across independent datasets. FISHNET integrates gene-level P-values with networks and functional annotations, based on the principle that spurious signals are randomly scattered, while true signals tend to cluster in functionally coherent subnetwork. Combining network topology, functional enrichment, and permutation-derived thresholds, FISHNET identifies a focused set of exceptional candidates termed FISHNET genes.

We used the whole blood tissue-specific METANet as the reference network input to FISHNET. This network was partitioned into modules using a network modularization algorithm based on modularity optimization. As input, we provided gene-level P-values derived from measured TWAS of 11 traits associated with cardiovascular risk in the Long Life Family Study (LLFS) cohort and performed replication analysis in the Framingham Heart Study (FHS) cohort [37, 38]. Across the 11 traits, FISHNET identified 114 FISHNET genes of which 24 were replicated. Using the whole blood tissue-specific PANDA network as input, we identified 0 FISHNET genes (see Additional file 2: Tables S1 and S2).

A central biological story emerging from these results centers on *SREBF2*, a replicated FISHNET gene negatively associated with HDL. Our findings support a model in which high HDL levels may suppress *SREBF2* activity, whereas low HDL permits *SREBF2* activation and sustains inflammatory signaling. *SREBF2* encodes SREBP2, a master transcription factor of sterol metabolism, canonically activated in adipocytes under low-cholesterol conditions [39–41]. Recent studies have shown that inflammatory stress can also activate SREBP2, enabling it to bind and induce inflammatory and interferon-response genes [42–44]. In the whole blood tissue-specific METANet, *SREBF2* appears in the same module as other negatively HDL-associated FISHNET genes–*S100A9, S100A12, TLR8,* and *ITGB2*–all of which converge on pathways that activate the NADPH oxidase 2 (NOX2) complex. *S100A9* and *S100A12* are calgranulins, a type of alarmin, that can act as ligands for the receptor for advanced glycation end products (RAGE), triggering assembly and activation of the NOX2 complex [45–47]. *TLR8* is a pattern recognition receptor that primes NOX2 activation in human neutrophils [48]. *ITGB2* promotes leukocyte adhesion to the endothelium and triggers “outside-in” signaling that further promotes NOX2 assembly and activation [49]. Collectively, these genes link *SREBF2* to NOX2-based excessive production of reactive oxygen species (ROS), which oxidize HDL and convert it into a pro-inflammatory form (oxHDL) that contributes to atherosclerosis. Notably, *S100A9*, *S100A12*, and *TLR8* also activate NF-κB, inducing cytokines (TNFα and IL-1β) known to activate *SREBF2* [50–53]. Together, these findings suggest a positive feedback loop in which reduced HDL allows *SREBF2* activation, which in turn promotes inflammatory cascades that further lower HDL, providing a mechanistic link between cholesterol metabolism, immune activation, and chronic low-grade inflammation characteristic of obesity.

## Discussion

In this work, we developed METANets, a supervised ensemble framework that combines TF motifs, cis-regulatory element (CRE) activity, and expression-derived features to construct tissue-specific TF network maps across 36 human tissues. Our modeling approach relied on three fundamental ideas: (1) using gene expression data (functional) to predict TF binding events (direct) prioritizes direct, functional targets of TFs, (2) incorporating both tissue-aggregate and tissue-specific expression features captures both the shared and tissue-specific regulatory edges, and (3) using tree-based regression models to incorporate both linear and nonlinear expression patterns improves model robustness. Our evaluations across multiple orthogonal metrics show that our approach significantly outperforms motif-only, expression-only, and unsupervised integrative methods in capturing direct and functionally coherent TF-TG relationships.

Gene expression-based models are adept at identifying functionally coherent gene sets, but the expression of a TF and a potential target gene could be correlated for reasons other than the TF regulating the gene directly: the TF might regulate indirectly (mediated by other proteins), or the TF and the gene might simply be co-regulated by a third factor. Conversely, motif-based approaches anchor predictions in physical binding potential but cannot confirm functional relevance, as potential binding sites are frequently unoccupied [6, 7] and even occupied sites often do not affect transcription [3–5, 54, 55]. By training our models to explicitly predict TF ChIP-seq binding events, METANets leverage the strengths of both data types without a noticeable tradeoff. Our results demonstrate METANets consistently outperforming the motif-only Marbach networks and our expression-only ETANets in the GO-directness metric that jointly assess directness and functional coherence.

METANets also illustrate the power of incorporating diverse feature types to model the complexity of gene regulation. We found that regulatory relationships are not exclusively linear or non-linear; by combining features from both LASSO (linear) and BART (non-linear) regression models, METANets captured a more direct and functionally coherent set of TF targets. Similarly, we that found neither tissue-specific nor tissue-aggregate features clearly outperformed the other in isolation, but together they predicted more robust sets of TF targets. The METANets approach performed best in the individual Binding and GO metrics without sacrificing GO-directness as a tradeoff. The practical utility of this approach was highlighted in our downstream genetic analysis, where the whole blood METANet enabled FISHNET to identify replicated gene-trait associations, proposing a module linking *SREBF2* to HDL and chronic inflammation.

Our work presents a subtle methodological shift for the field of regulatory network inference. For years, the community has largely relied on two distinct categories of methods: those based on physical binding evidence like TF motifs, which often lack functional validation, and those based on gene expression, which are susceptible to confounding by indirect relationships. While unsupervised integration methods have attempted to bridge this gap, METANets presents a new alternative by successfully implementing a supervised learning framework. The core innovation is reframing the problem: instead of merely correlating different data types, we use functional data (gene expression) to predict direct physical interactions (TF binding).

This approach provides a methodological foundation for using the ever-expanding space of omics data integration. The principle of using one data modality as a ground-truth label to train predictive models on other, more widely available data types can be generalized beyond the specific application in this work. It makes TF network mapping more broadly applicable, as ChIP-seq data for every TF is not available, while RNA-seq data is abundant. Our approach can make use of ChIP-seq in predicting targets of TFs that have ChIP-Seq data, but it can also extract general patterns in gene expression data that are predictive of direct binding and use those patterns to infer targets for TFs that do not have ChIP-Seq data. We also presented a new evaluation of networks based on utility for applications rather than network characteristics.

Several limitations suggest avenues for further development. One key limitation is the lack of tissue-specific TF binding data in our framework. While tissue-specific expression data are readily available at scale, obtaining comparable TF binding data across tissues remains challenging. As a result, METANets relied on tissue-aggregated TF binding data. Emerging paired-seq technologies that jointly measure TF binding and gene expression in the same cell could make predictions of TF binding events more robust and the single-cell measurements can facilitate the direct mapping of granular cell-type-specific networks. Development of scalable methods to address the inherent sparsity of single-cell data will be crucial for realizing this goal.

Another opportunity lies in extending the METANets framework to additional omics data types. Chromatin accessibility (ATAC-seq) and chromatin conformation (Hi-C, HiChIP) represent rich sources of contextual information that could further refine predictions of TF-TG relationships. Beyond tissues, the same framework could be adapted to disease states, environmental exposures, or dynamic perturbations, producing condition-specific network maps that illuminate regulatory mechanisms underlying phenotypic variation.

In summary, METANets advance the state of regulatory network mapping by demonstrating that supervised integrative approaches can accurately prioritize direct, functional TF-TG interactions. Beyond their immediate utility, METANets highlight the potential of combining heterogeneous data sources to produce context-dependent regulatory maps, setting the stage for increasingly precise models of human gene regulation.

## Methods

### Transcription factor (TF) binding data

TF binding locations were determined using data from TF ChIP-seq (Chromatin Immunoprecipitation followed by sequencing) experiments. We downloaded TF ChIP-seq data from the REMAP database, which consists of 5,798 experiments for 1,135 TFs across over 600 tissues and cell lines [28]. TFs that did not meet the criteria defined by Lambert et al. [56] were removed. Additionally, binding peaks with FDR q-value ≤ 0.01 were removed for each TF. After these filtering steps, we retained 799 TFs with high-confidence binding peaks.

### Regulatory element annotations

Promoter and enhancer annotations were obtained from the FANTOM5 database, which contains data on 209,911 promoters and 41,456 enhancers for 17,778 protein-coding genes, along with activity level measurements across 1,829 cell, tissue, and cell-line samples [32, 33].

### Mapping binding peaks to regulatory regions

For each TF, all high-confidence (FDR q-value ≤ 0.01) binding peaks within FANTOM5 promoter and enhancer regions were examined. In cases where multiple overlapping peaks were present within a single regulatory element, the peak with the highest confidence score among the overlapping regions was retained. A score was calculated for each regulatory element by summing the confidence scores of the retained peaks, weighted by their length in base pairs (see Additional file 1: Fig. S2). For each gene, a gene score was derived by aggregating the scores of all regulatory elements annotated for the gene. Since TF binding events within each regulatory region represent independent evidence of gene regulation, the aggregated score across regulatory elements reflects the cumulative likelihood of the TF regulating the gene.

### Marbach networks

We downloaded 394 cell-type- and tissue-specific networks from Marbach et al. [23]. We formed 36 tissue networks by merging closely related networks by taking the graph union (union of the node and edge sets while retaining the maximum weight for each edge). We used the mapping file for GTEx eQTL validation from Marbach et al. as the baseline reference for 13 GTEx tissues. We manually curated the remaining 23 tissues using annotations from the BRENDA tissue ontology [57] (see Additional file 2: Table S3).

### Tissue-specific gene expression data

We downloaded bulk RNA-seq read counts and transcripts per million (TPM) data from the Genotype-Tissue Expression (GTEx) consortium V8 release [58]. The GTEx project provides a comprehensive catalogue of gene expression profiles across 54 human tissues using biospecimens from approximately 1,000 postmortem donors. The GTEx read count data for protein-coding genes was filtered to remove lowly expressed genes. Specifically, genes with ≤ 3 counts per million in ≥ 98.5% of samples were removed. Genes that failed to pass this filter were also removed from the TPM data.

### Construction of binding labels

The network mapping approach uses binary labels representing binding events for each TF-gene pair. We assigned a positive label to genes with nonzero binding scores for each TF to construct binary labels for TF-gene binding events. If more than 10% of genes had a positive score for a given TF, only the top 10% of highest-scoring genes were retained as positive labels. All remaining gene pairs were labeled as negative.

### Construction of gene expression-based features

The gene expression-based features were constructed by training two regression algorithms, LASSO and Bayesian Additive Regression Trees (BART), to predict the expression level of each gene from the expression levels of all TFs [11–13, 59]. LASSO (Least Absolute Shrinkage and Selection Operator) is a linear regression method with L1 regularization. BART is a non-parametric sum-of-trees model that captures complex, non-linear relationships through an ensemble of regression trees. Features for a given TF-gene pair reflect the effect of the TF’s expression level on the gene’s predicted expression level in the trained models – for example, the feature extracted from a LASSO model is the learned coefficient of the TF’s expression level. Both algorithms are run on the filtered tissue-specific gene expression TPM data and, separately, on the aggregated TPM data from all GTEx tissue samples, yielding 4 regression features for each TF-gene pair.

### Training Machine Learning Algorithms

METANets were inferred by combining diverse features using the XGBoost model [60]. **Error! Reference source not found.**A depicts the inputs and outputs of the reconstruction of METANets. A separate XGBoost model was trained for each of the 36 tissues, where instances are TF-target gene (TG) edges for a specific tissue. This machine learning model employs gradient-boosted decision trees for regression and classification tasks. The Python XGBClassifier implementation (v2.1.1) was used, with the binding labels as the response variable and a combination of tissue-specific and tissue-aggregate features as predictors. Tissue-specific features include TF-TG edges from the Marbach networks and edges inferred from LASSO and BART. Tissue-aggregate features include edges inferred from LASSO and BART using samples from all tissues.

The models were trained using 10-fold nested cross-validation, stratified based on the distribution of binding labels. A new XGBoost model was trained for each outer fold, using the remaining 9 folds as training data and the single fold a test data. The training data within each outer fold was further divided into 5 inner folds for hyperparameter tuning. Hyperparameter tuning was conducted using the Tree of Parzen Estimators (TPE) from the Hyperopt package (v.0.2.7), which models hyperparameters as nodes in a tree structure [61, 62]. The predicted probabilities that the TF binds in the gene’s CREs, in the test folds, are used as edge scores in our tissue-specific METANets.

### Binding metric

We calculated the percentage of top-scoring TF-TG edges supported by TF binding data at different thresholds. Top-scoring edges were defined by sorting edges by the edge score in descending order. Thresholds were scaled to the number of target genes per TF. This is an average, so different TFs have different numbers of target genes at each threshold. A higher percentage of binding support reflects greater enrichment of direct edges in the network map. The random expectation is the probability that a randomly selected TF-TG edge is supported by the binding data.

### GO metric

We performed GO enrichment analysis for each TF’s targets using GO-Term-Finder v0.86 [63]. To focus on terms associated with specific biological processes, rather than extremely generic terms like ‘biosynthetic process’, we excluded terms with number of annotated genes below 5 or exceeding 300. Each TF’s score was the maximum −log P-value over all GO terms. If a TF’s target set showed no significant enrichment for any GO biological process, we assigned the TF a P-value of 1 (equivalent to −log P = 0). The final network-level score was taken as the median of these scores across all TFs. We did these analyses for each threshold, as in the binding metric, but without excluding TFs that did not have binding data available.

### GO-directness metric

To determine whether improvements on the GO evaluation metric came at the cost of including indirect edges, we calculated, for each TF, the percentages of binding support for edges that matched the GO term with the highest minus log P-value. For each threshold, we reported the average percentage of binding support across TFs. The random expectation is identical to that of the Binding metric.

### PPI metric

First, we calculated the Jaccard similarity between the sets of target genes of each (TF, TF) pair; the Jaccard similarity is 1 when the target sets are the same and 0 when none of the targets in the sets are shared. Next, we sorted (TF, TF) pairs by Jaccard similarity and calculated the percentage of the top 100 pairs that are supported by physical interactions from the protein-protein interaction (PPI) STRING database[34]. The database has 477,903 interactions predicted with high confidence (SRTING score ≥ 0.7). A higher percentage of PPI support means that (TF, TF) pairs are likely to work as a part of a known physical protein complex. We did these analyses for the TF-TG edges at thresholds of 50 and 100 targets per TF. The random expectation is the probability that a randomly selected pair of TFs present in a tissue network is supported by the PPI database. This probability was computed separately for each tissue and then averaged across the 36 tissues.

### Empirical Null for Network Quality

To establish an empirical null to evaluate network quality, we generated 50 permuted networks by randomly shuffling the fully connected METANet edge scores. Each permuted network was subjected to the same cutoff thresholds as the real networks and evaluated across all four metrics, providing a consistent null distribution.

### Processing of PANDA Networks

Gene regulatory networks for 38 tissues were downloaded from Zenodo [64]. To enable direct comparison with our METANets, we retained the 26 tissues shared with the 36 METANet tissues. For each tissue, we used two versions of the PANDA networks: (1) the full networks containing weighted edges between all transcription factors (TFs) and genes, and (2) the tissue-specific networks provided in the same dataset, in which tissue-specific edges had already been annotated by the original authors. To maintain consistency, both versions of the PANDA networks were subjected to the same downstream processing steps: restricting to protein-coding gene targets, removing self-regulatory edges, and retaining only the subset of TFs common to both PANDA and METANets.

### Tissue-Specific Edge Filtering

Tissue-specific regulatory edges were identified using the interquartile range (IQR)-based filtering approach originally introduced by Sonawane et al. [20]. For each TF-TG pair (i,j), the edge weight in tissue t is denoted $w_{ij}^{\left(t\right)}$. For each edge, we computed a normalized specificity score by comparing the tissue-specific weight to the distribution of weights across all tissues:

$$s_{ij}^{\left(t\right)}=\frac{w_{ij}^{\left(t\right)}\;-\;median\left(w_{ij}\right)}{IQR\left(w_{ij}\right)},$$

where $median\left(w_{ij}\right)$ and $IQR\left(w_{ij}\right)$ are calculated across all tissues for the same edge. Edges with $s_{ij}^{\left(t\right)}>2$ were considered specific to tissue t. This filtering approach was applied to METANets to construct tissue-specific versions of our networks, directly comparable to the tissue-specific PANDA networks generated in Sonawane et al. [20].

### Validation of Tissue Specificity using eQTL Data

We downloaded cis-eQTL data from the GTEx consortium V8 release [31]. Significant variant-gene associations were obtained for each of the 36 tissues. For each TF-target gene (TF-TG) edge in a network, we determined whether one or more of a tissue’s significant variants overlapped the TF’s ChIP-seq peaks within the promoter or annotated enhancers of the target gene. We defined two complementary metrics for a given network:

1. eQTL support: the fraction of edges supported by at least one tissue-specific eQTL.
2. eQTL count: the sum, across all edges, of the number of tissue-specific eQTLs supporting each edge.

To assess a network’s tissue-specificity, we performed a permutation test. First, we calculated an observed statistic $S_{\mathrm{obs}}$ by ranking all 36 networks within each tissue and summing the ranks of the “matching” pairs (e.g., the rank of the liver network in the liver tissue). A smaller sum indicates better overall specificity. Next, to generate a null distribution, we performed 10000 permutations. In each permutation, we randomly shuffled the network labels before summing the ranks of the newly assigned matching pairs to obtain the permutation statistic $S_{\mathrm{perm}}$. This process simulates the null hypothesis that there is no true correspondence between a network and its native tissue. The empirical p-value was then calculated as the proportion of permutations with $S_{\mathrm{perm}}\leq S_{\mathrm{obs}}$ (see Additional file 1: Fig. S4)

Direct comparisons between different networks were performed using the Wilcoxon signed-rank test on the distributions of matching ranks.

### Gene-trait Association Analysis using FISHNET

We applied FISHNET, a network-guided replication framework that integrates prior biological knowledge with gene-level association statistics, to evaluate the biological relevance of tissue-specific METANets [65]. FISHNET is designed to prioritize genes that exhibit suggestive association signals but are more likely than expected by chance to replicate across independent datasets.

The primary input to FISHNET was the whole blood tissue-specific METANet. For comparison, we also applied FISHNET using the whole blood tissue-specific PANDA network constructed by Sonawane et al. (2017). Each network was partitioned into modules using the and M1 modularity optimization algorithm, as implemented in the MONET toolbox [66].

As input gene-level signals, we used transcriptome-wide association study (TWAS) p-values derived from the Long Life Family Study (LLFS). We considered 11 traits associated with cardiovascular risk, including body mass index (BMI), high-density lipoprotein (HDL), low-density lipoprotein (LDL), and triglycerides, among others. Replication was assessed using gene-level association statistics from the Framingham Heart Study (FHS) cohort, analyzed for the same 11 traits. Details on RNA-seq data processing and covariate adjustments have been previously described by Acharya et al. [65].

## Supplementary Material

Supplement 1

Supplement 2

## Figures and Tables

**Table 1:** Tissue specificity evaluation of METANet and Marbach against the null across 36 tissues.

Empirical test for tissue-specificity for 36 tissues			
eQTL support	Average number of targets per TF		
	50	100	250
METANet	0.498 (666)	**0.027 (597)**	**0.022 (613)**
Marbach	**0.003 (567)**	**2×10^−4^ (554)**	**6×10^−4^ (537)**
eQTL counts	Average number of targets per TF		
	50	100	250
METANet	0.106 (604)	**0.003 (535)**	**0.007 (563)**
Marbach	0.119 (611)	0.173 (623)	**3.4×10^−3^ (537)**

Permutation test p-values (and the sum of observed matching ranks) against the distribution of 10000 permutation sums. METANets showed significant tissue specificity compared to the null in eQTL support at average 100 and 250 targets per TF thresholds. The Marbach networks showed significant tissue specificity in eQTL support at all thresholds. METANets showed significant tissue specificity in eQTL counts at average 50 and 100 targets per TF thresholds. The Marbach networks show significant tissue specificity in eQTL counts at average 250 targets per TF only. See also Additional file 1: Fig. S1.

**Table 2:** Tissue specificity evaluation of METANet and PANDA against the null across 26 tissues.

Empirical test for tissue-specificity for 26 tissues			
eQTL support	Average number of targets per TF		
	50	100	250
METANet	0.241 (333)	0.096 (320)	0.143 (324)
PANDA	0.091 (319)	0.176 (330)	0.424 (346)
eQTL counts	Average number of targets per TF		
	50	100	250
METANet	0.297 (335)	**0.007 (284)**	0.129 (324)
PANDA	0.202 (329)	0.245 (331)	0.183 (328)

Permutation test p-values (and the sum of observed matching ranks) against the distribution of 10000 permutation sums. METANets and PANDA networks did not show significant tissue specificity in eQTL support. METANets were significantly tissue specific in eQTL counts at the average 100 targets per TF threshold. PANDA networks remained not significantly tissue specific in eQTL counts. See also Additional file 1: Fig. S1.

**Table 3:** Tissue specificity evaluation against Marbach.

Wilcoxon signed-rank test vs. Marbach			
Average number of targets per TF	50	100	250
eQTL support	0.95 (294.0)	0.30 (266.5)	0.88 (289.0)
eQTL counts	0.24 (243.0)	0.84 (269.5)	0.43 (282.0)

Wilcoxon signed-rank test p-values (and W statistics) between matching tissue ranks of METANets and Marbach networks for 36 tissues. Across all thresholds of average number of targets per TF, METANets and Marbach networks were not significantly different.

**Table 4:** Tissue specificity evaluation against PANDA.

Wilcoxon signed-rank test vs. PANDA			
Average number of targets per TF	50	100	250
eQTL support	0.57 (152.0)	0.78 (152.0)	0.87 (156.5)
eQTL counts	0.90 (145.5)	0.41 (132.0)	0.88 (144.5)

Wilcoxon signed-rank test p-values (and W statistics) between matching tissue ranks of METANets and PANDA networks for 26 tissues. Across all thresholds of average number of targets per TF, METANets and PANDA networks were not significantly different.

**Table 5:** Empirical test of tissue-specific METANet and tissue-specific PANDA against the null across 26 tissues.

Empirical test for tissue-specificity for 26 tissues		
Network	eQTL support	eQTL count
Tissue-specific METANet	**0.034 (307)**	0.250 (338)
Tissue-specific PANDA	0.233 (332)	0.199 (341)

Permutation test p-values (and the sum of observed matching ranks) against the distribution of 10000 permutation sums. Tissue-specific METANets showed significant tissue specificity in eQTL support and tissue-specific PANDA networks were not significantly tissue-specific. Neither tissue-specific METANets nor tissue-specific PANDA networks were significantly tissue-specific in eQTL counts. See also Additional file 1: Fig. S1.

**Table 6:** Tissue specificity evaluation against tissue-specific PANDA.

Wilcoxon signed-rank test vs. tissue-specific PANDA	
eQTL support	0.485 (125.0)
eQTL counts	0.881 (168.5)

Wilcoxon signed-rank test p-values (and W statistics) between matching tissue ranks of tissue-specific METANets and tissue-specific PANDA networks for 26 tissues. Full tissue-specific networks were evaluated, without thresholding for top-scoring edges. No significant difference between tissue-specific METANets and tissue-specific PANDA in both eQTL support and eQTL count.

## Data Availability

METANets, tissue-specific METANets, as well as other networks evaluated in this work are available at https://doi.org/10.5281/zenodo.17309371. Code is available at https://github.com/BrentLab/METANet. All data for modeling were downloaded from the publicly available sources indicated in Methods.
