# Supplementary material for: Combining Motifs, CRE Activity, And Gene Expression Data Using ML Greatly Improves the Accuracy of Tissue-Specific TF Network Maps
Source: bioRxiv. 2025 Oct 13:2025.10.10.681634. Preprint. [Version 1] doi: 10.1101/2025.10.10.681634 (PMC12632818; doi:10.1101/2025.10.10.681634)
Supplement: Supplement 1 [file media-1.pdf]

Supplementary Figures

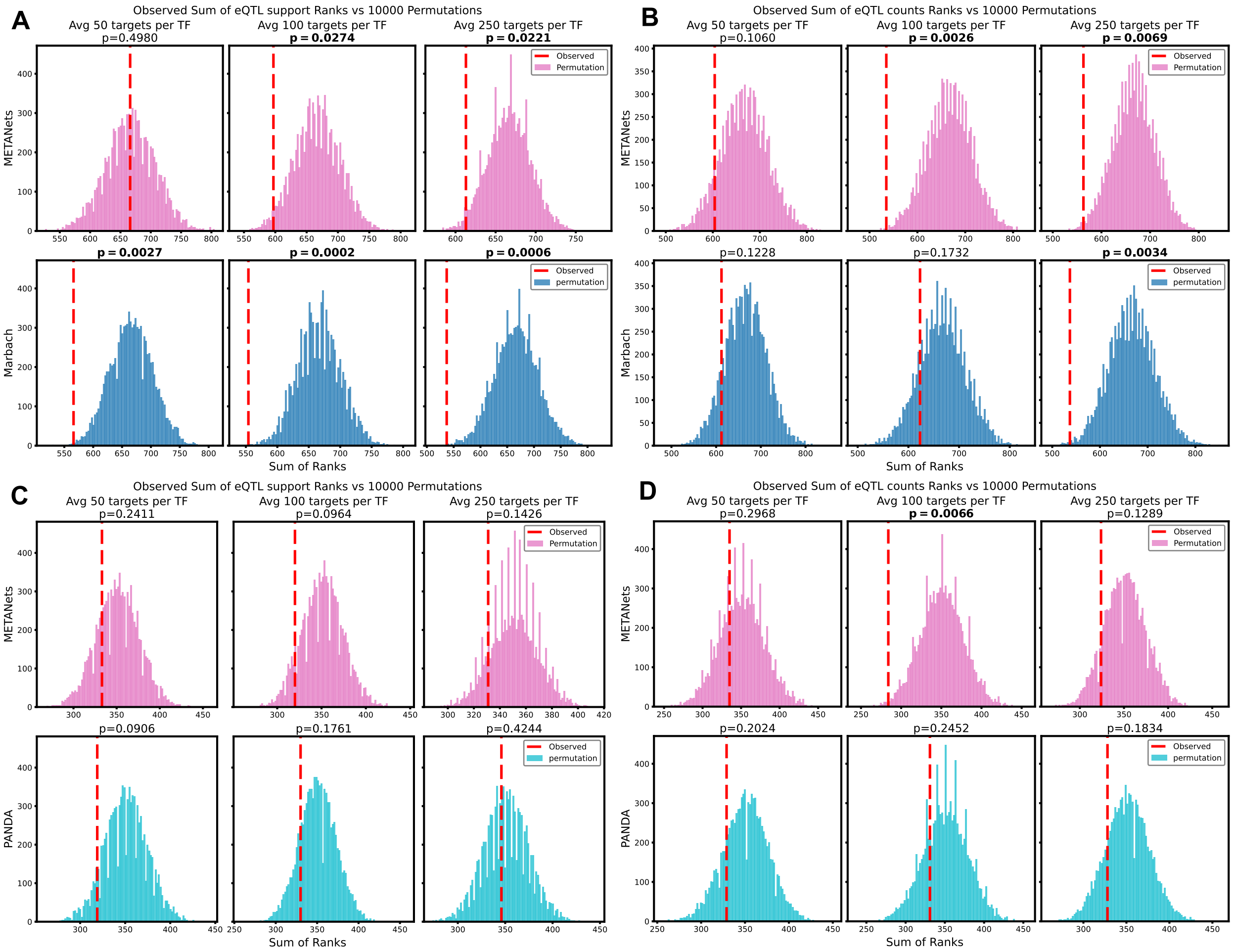

Figure S1 Tissue specificity evaluation against the empirical null. The sum of eQTL support or eQTL counts ranks (dashed red line) plotted against a null distribution of 10000 sums of permuted ranks. METANets and Marbach networks were evaluated across 36 tissues for (**A**) eQTL support and (**B**) eQTL counts. METANets and PANDA networks in evaluated across 26 tissues for (**C**) eQTL support and (**D**) eQTL counts. METANets have tissue specificity comparable to other networks, except that Marbach networks have better eQTL support at average 50 targets per TF (panel A).

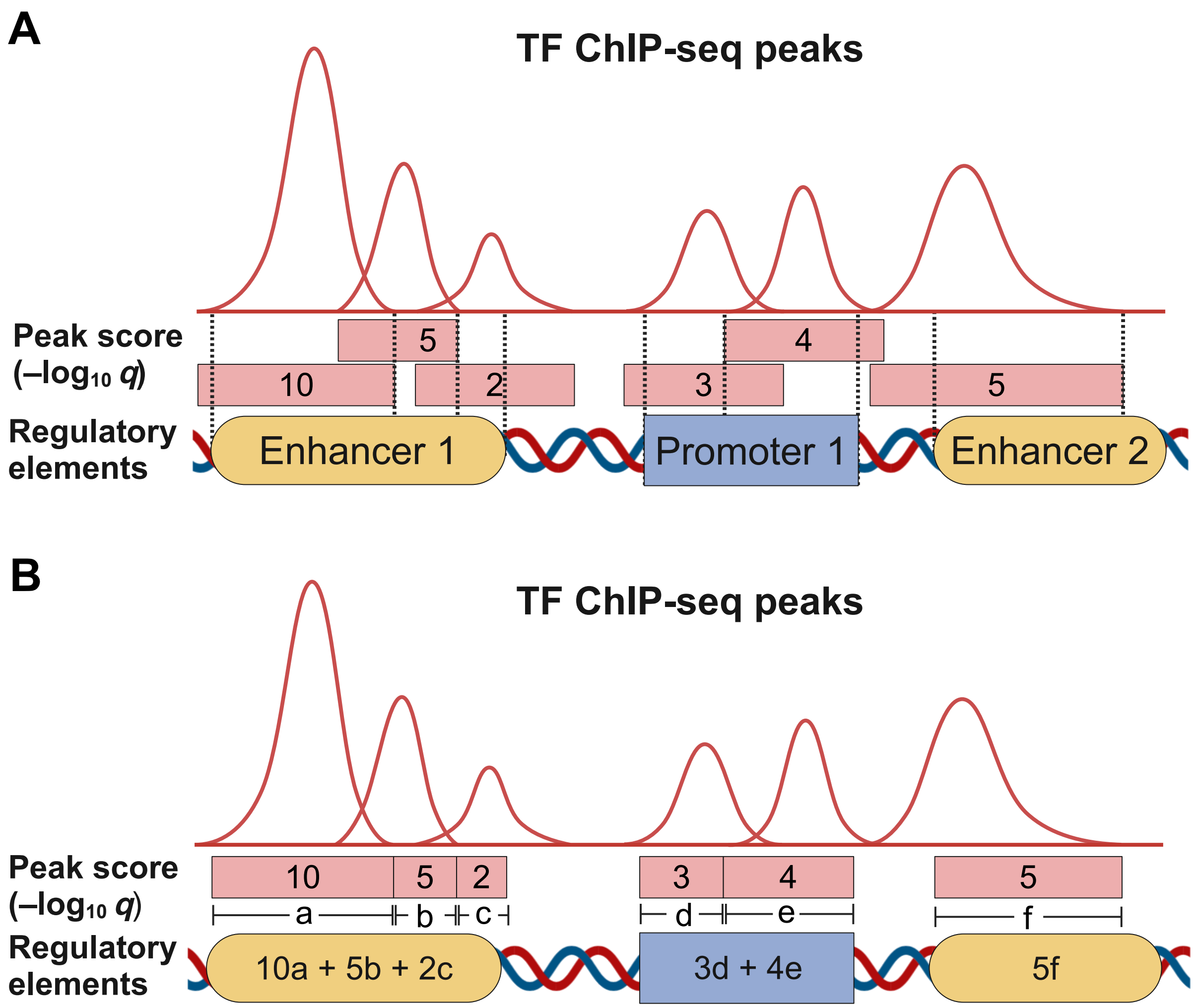

Figure S2 Example illustration of assigning TF binding scores to FANTOM5 regulatory elements. (A) Multiple TF ChIP-seq peaks may overlap in a single regulatory element. Dotted vertical lines indicate cutoffs for overlapping peaks, retaining the peak with maximum peak. (B) Each regulatory element's score is calculated as the sum of retained peak scores weighted by the peak region length.

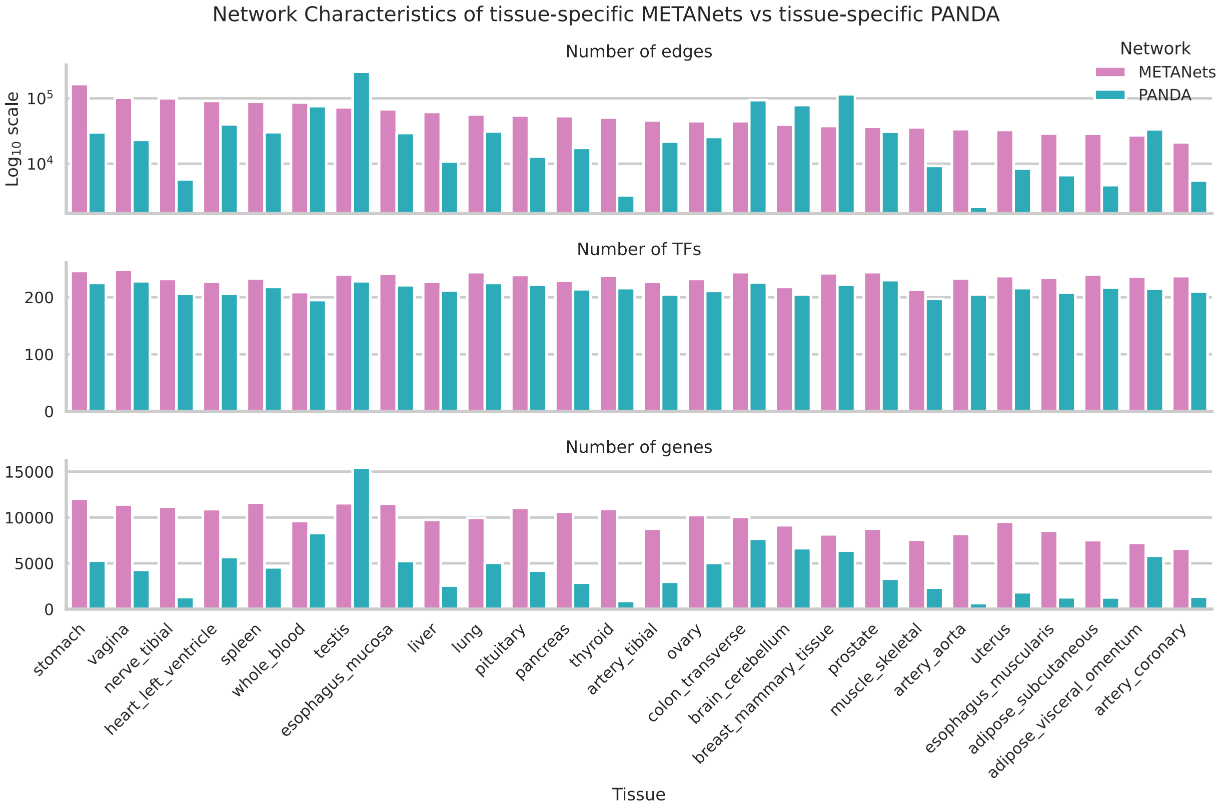

Figure S3 Network characteristics of tissue-specific METANets and tissue-specific PANDA networks PANDA networks were first restricted to protein-coding genes, with self-regulatory edges excluded, and subsequently subsetted to TFs present in the corresponding tissue's METANet. See Additional file 2: Table S4 for tabulated details of this figure.

Original ranks		Network tissue			
		Brain	Liver	…	Blood
eQTL tissue	Brain	**2**	3	…	9
	Liver	4	**13**	…	7
	⋮	…	…	**…**	…
	Blood	31	36	…	**1**

→

Permutation ranks		Permuted network tissue			
		Blood	Brain	…	Liver
eQTL tissue	Brain	**9**	2	…	3
	Liver	7	**4**	…	13
	⋮	…	…	**…**	…
	Blood	1	31	…	**36**

Figure S4 Permutation to generate empirical null distribution of ranks For each tissue's eQTL data, all tissue networks are ranked based on either the eQTL support or eQTL counts metric, breaking ties using the other metric. Each diagonal entry in the original ranks table represents the rank of the "matching" network for the given tissue's eQTL. The sum of the diagonal entries in the original ranks table (left) is the observed statistic. For each of 10000 permutations, we shuffle the columns of the table. The sum of the diagonal entries in this shuffled table is the permuted statistic. This process simulates the null hypothesis that there is no true correspondence between a network and the tissue it was constructed for.

Supplementary Note 1. Tissue-specific network filtering

Tissue-specific regulatory edges were identified using the filtering approach originally introduced by Sonawane et al. [1]. Each tissue METANet varies in total edge count due to differences in gene expression coverage—some genes fail to pass tissue-specific expression filters and thus lack corresponding edges in certain tissues. Because the tissue-specificity score $s_{ij}^{(t)}$ depends on computing both the median and the IQR of edge weights w_{ij} across all tissues, edges missing in many tissues results in too few observations to reliably estimate their across-tissue variability. We therefore excluded edges absent in more than 12 of the 36 tissues. This step retained 77.16% of the complete edge set.

Supplementary Note 2. Module Identification for FISHNET

Network modules were identified using the top three algorithms from the Disease Module Identification, DREAM Challenge [2]: K1 (kernel clustering), M1 (modularity optimization), and R1 (random walk-based clustering). We implemented these algorithms through the MONET package [3] and applied each to the whole-blood-specific TF network maps from METANet and PANDA. The M1 algorithm was run in both directed and undirected modes by varying the "--linksdir" parameter. Summary characteristics of the modules generated by each algorithm and network are provided in Additional file 2: Table S1. The final modules used for the *SREBF2*-related biological analysis were derived from the directed M1 algorithm.

REFERENCES

1. Sonawane AR, Platig J, Fagny M, Chen C-Y, Paulson JN, Lopes-Ramos CM, DeMeo DL, Quackenbush J, Glass K, Kuijjer ML: **Understanding Tissue-Specific Gene Regulation**. *Cell Reports* 2017, **21**(4):1077-1088.
2. Choobdar S, Ahsen ME, Crawford J, Tomasoni M, Fang T, Lamparter D, Lin J, Hescott B, Hu X, Mercer J: **Assessment of network module identification across complex diseases**. *Nature methods* 2019, **16**(9):843-852.

3. Tomasoni M, Gómez S, Crawford J, Zhang W, Choobdar S, Marbach D, Bergmann S: **MONET: a toolbox integrating top-performing methods for network modularization**. *Bioinformatics* 2020, **36**(12):3920-3921.
